# Parallel reductions of IgE and exhaled nitric oxide after optimized anti‐inflammatory asthma treatment

**DOI:** 10.1002/iid3.103

**Published:** 2016-03-21

**Authors:** Jörgen Syk, Andrei Malinovschi, Magnus P. Borres, Anna‐Lena Undén, Anna Andreasson, Mats Lekander, Kjell Alving

**Affiliations:** ^1^ Department of NeurobiologyCare Sciences and Society, Karolinska InstitutetStockholmSweden; ^2^ Centre for Allergy Research, Karolinska InstitutetStockholmSweden; ^3^ Department of Medical SciencesClinical Physiology, Uppsala UniversityUppsalaSweden; ^4^ Department of Women's and Children's HealthUppsala UniversityUppsalaSweden; ^5^ Stress Research InstituteStockholm UniversityStockholmSweden; ^6^ Department of Clinical NeuroscienceKarolinska InstitutetStockholmSweden

**Keywords:** Asthma, asthma control, atopy, breath test, corticosteroid, F_E_NO, immunoglobulin E, leukotriene‐receptor antagonist, quality of life

## Abstract

Immunoglobulin E (IgE) is crucial for the development of airway inflammation in atopic asthma, and inhibition of IgE using monoclonal antibodies is now part of asthma therapy. However, the impact of ordinary anti‐inflammatory treatment on IgE is unclear. The aim of this study was to investigate if optimization of treatment with inhaled corticosteroid (ICS) and leukotriene‐receptor antagonist (LTRA) according to symptoms or exhaled nitric oxide (F_E_NO) levels over a one‐year period affects IgE concentrations. Altogether, 158 relatively well‐controlled but multi‐sensitized asthmatics (age 18–65 years), with ongoing ICS treatment at baseline, were included in this post hoc analysis of data from a randomized, controlled trial on F_E_NO‐guided asthma therapy. Asthma control and quality of life (Juniper ACQ and mAQLQ), F_E_NO, and serum IgE were measured at baseline and after one year. Concentrations of IgE antibodies to six common perennial aeroallergens were summed up (perennial IgE). We found that perennial and total IgE decreased by 10.2% and 16.0% (*P* < .001 both comparisons). This was not related to allergen exposure, whereas the total use of ICS and LTRA during the year correlated with the reduction in perennial IgE (*P* = .030 and *P* = .013). The decrease in perennial and total IgE correlated significantly with the reduction in F_E_NO (*P* < .003 and *P* < .001), and with improvements in ACQ and mAQLQ scores (*P* < 0.05, all comparisons). We conclude that one year of optimization of treatment with ICS and LTRA in patients with persistent atopic asthma resulted in significant decreases in total IgE and IgE antibodies; these decreases correlated with a reduction in F_E_NO and improvements in asthma control and quality of life. Thus, IgE is reduced by ordinary asthma controller medications and the effect on IgE seems to be clinically important.

## Introduction

Asthma is by definition an inflammatory disorder of the airways. Immunoglobulin E (IgE) is often involved in the inflammation, as the majority of asthmatics have atopic asthma, which is most commonly initiated in childhood by sensitization to airborne allergens 1. Asthma prevalence has also been shown to correlate directly with increased levels of total IgE, regardless of proven atopy 2, 3. The airway inflammation in atopic asthma is recognized as Th2 lymphocyte‐driven and Th2 cytokines modulate the inflammation with, for example, increased IgE concentration, elevated fraction of exhaled nitric oxide (F_E_NO), and recruitment and activation of eosinophilic granulocytes and mast cells 1, 4, 5. Inhaled corticosteroids (ICS) represent the most effective maintenance therapy for this group of patients. Humanized monoclonal antibodies against IgE are used for treatment of moderate to severe atopic asthma 1, 6; thus, a reduction of IgE concentration has potential to reduce asthma symptoms and improve management of asthma.

Reports on the effect of corticosteroid treatment on IgE concentrations in patients with atopic asthma have been equivocal and few long‐term studies have been done. There are unanimous reports of an initial transient increase of IgE during the first 1–2 weeks of treatment with systemic corticosteroids 7, 8, 9. Two small studies show that the introduction of at least a medium dose of ICS lowers IgE in corticosteroid‐naïve patients 10, 11. However, long‐term effects are unclear 12.

In this post hoc analysis of a randomized controlled trial of F_E_NO‐guided asthma therapy (the NOAK study), we merged data from two study groups (F_E_NO‐guided or symptom‐guided asthma therapy) for analysis of one‐year follow‐up data 13. The aim was to investigate if a period of optimization of anti‐inflammatory treatment could affect IgE concentrations in atopic asthma patients with ongoing ICS treatment at baseline. We also wanted to study if changes in IgE were related to treatment intensity or changes in allergen exposure. Further, we aimed to analyze the relation between changes in IgE and markers of Th2‐driven inflammation, as well as possible effects of changes in IgE on asthma control and quality of life.

## Methods

### Study design and participants

All data in this study were collected from the study *Optimisation of anti‐inflammatory asthma treatment using exhaled NO to improve asthma‐related quality of life within primary health care* (the NOAK study), which was an open‐label, parallel‐group, randomized, controlled study, conducted at 17 primary health care centers in central and southern Sweden from November 2006 to March 2010 13. A total of 187 participants were recruited; 181 came to the baseline visit and were randomized to F_E_NO‐guided treatment or control group (usual care) and were followed up at four visits over a period of one year. Those who had donated blood samples both at baseline and at the last visit of the study (*n* = 158) were included in this analysis. Eligible participants had a doctor's diagnosis of asthma, had been on prescribed ICS treatment for at least the past six months, and had confirmed IgE sensitization to at least one major airborne perennial allergen (dog, cat, or mite). Participants were 18–65 years old, non‐smokers for at least the past year, and with a smoking history of <10 pack‐years. For the F_E_NO‐guided group, the anti‐inflammatory treatment was adjusted using an algorithm based on F_E_NO 13. In the control group, the F_E_NO measurement was double‐blind and treatment was adjusted according to routine clinical practice (usual care). ICS and LTRA were used as anti‐inflammatory treatment and participants in both groups were placed in one of six fixed treatment steps, where LTRA was added at the two highest steps (Table S1). At every visit, the following data were registered: F_E_NO, self‐reported exposure to pets, and dampness in the home (window pane condensation), asthma control measured with Elizabeth Juniper's Asthma Control Questionnaire (ACQ), and LTRA and ICS use (budesonide equivalents) 14. Asthma‐related quality of life, measured with Elizabeth Juniper's mini Asthma Quality of Life Questionnaire (mAQLQ), and general quality of life, measured with the Gothenburg Quality of Life Instrument (GQLI), were registered at three visits, and spirometry was done at the start and end of the study (see online repository for more information) 15, 16. Venous blood was sampled for analysis of IgE antibodies, total IgE and IgG_4_‐antibody concentration, and serum eosinophilic cationic protein (S‐ECP) at the start and end of the study (See Table S2 for a study design summary). IgE, IgG_4_, and ECP were analyzed in a Phadia^®^ 100 system with ImmunoCAP^®^ reagents (Immunodiagnostics, Thermo Fisher Scientific, Uppsala, Sweden). IgE antibodies measured = *Dermatophagoides pteronyssinus*, *Dermatophagoides farina*, cat, dog, horse, timothy, birch, *Cladosporium herbarum*, and mugwort. IgG_4_ antibodies measured = cat and timothy. Serum samples were initially stored at −20°C (<6 months) and then at −80°C. Samples from each patient from the start and end of the study were analyzed side‐by‐side in the instrument for all serum measurements. Pollen count data were collected during 2006–2009 from three stations, Stockholm, Forshaga, and Malmö, which were as close to the participating primary health care centers as possible (see online repository for more information).

The study was approved as a multi‐center study by the regional ethics committee in Stockholm (Dnr: 2006/185‐31). All participants provided written informed consent.

### Study rationale

IgE was a secondary endpoint in the original protocol. However, when analyzing the change in IgE, it was found that most IgE was reduced in both treatment groups, with no significant difference between groups (Table S3). Since a reduction in IgE has been rarely reported, we wanted to examine what variables associated with reduced IgE. For this post hoc analysis, the two groups were merged to increase the number of patients. Even though the treatment was adjusted on different grounds in the original study groups, both groups had to use the same treatment steps (Table S1). Mean ICS use did not change significantly within or between study groups during the study, but there was a significant redistribution of treatment steps in both groups 13. Thus, participants with elevated F_E_NO (≥24 ppb in women and ≥26 ppb in men)—or with more symptoms, depending on which group they belonged to—received more treatment, and participants with low F_E_NO or few symptoms received less treatment 13. The anti‐inflammatory treatment increased over the year in those with high baseline F_E_NO in both study groups, probably due to some degree of covariation of symptoms and F_E_NO (ACQ score and FeNO tended to correlate at baseline, *P* = .060 (Spearman test)). This resulted in a significant decrease in F_E_NO in subjects with elevated F_E_NO at baseline in both groups, with no significant difference between the groups (F_E_NO‐guided group: −14.3 ppb, *P* < .002, usual care group: −12.6 ppb, *P* < .006; *P* = .91 between groups). When results from the subjects with elevated baseline F_E_NO in the two study groups were analyzed together, this showed an increase in the proportion of participants with LTRA from 2.5% to 22.2% (*P* < .001), mean ICS use increased by 180 μg/day (*P* < .001), and mean F_E_NO decreased by 13.5 ppb (*P* < .001).

### Statistical methods

The concentrations of individual IgE antibodies were grouped and summed up. The groups were: *perennial* allergens (cat, dog, horse, mite × 2, and mold), *seasonal* allergens (birch, timothy, and mugwort), *food* allergens (fx5 = cow's milk protein, egg white, peanut, soy, wheat, and fish), and *all* allergens (perennial, seasonal, and food). Two‐sided tests with *P*‐values less than .05 were considered significant and indicated in bold in figures. Results were analyzed with Mann–Whitney *U*‐test for comparison between groups and Wilcoxon signed‐rank test for analysis of change within groups between first and last visit, or the corresponding Student's *t*‐tests. The effect of inclusion in different quarters of the calendar year was analyzed with Kruskal–Wallis test and corrected for multiple comparisons. Categorical data were analyzed with χ^2^ test, or Fisher's exact test when applicable. Spearman's rank test or Pearson's test were used for correlation tests. For IgE antibody measurements, values down to zero were used in the calculations. IgG4 and IgE data were converted to the log base 10 scale and analyzed using a paired *t*‐test when applicable. IgE values of zero were replaced by .001 before conversion to log base 10 scale. Statistical analyses were performed with Stata/IC11 version 11.2 (Stata Corp, 2009, College Station, TX). The study is registered with ClinicalTrials.gov (identifier NCT00421018).

## Results

Baseline characteristics and one‐year follow‐up data for included patients are presented in Table 1.

**Table 1:** Patient characteristics at baseline and last visit

	Baseline	One‐year follow‐up	*P*
Demographic characteristics			
Male	82/158 (51.9)	N.A.	
Age	41.2 (12.4)	N.A.	
Height (cm)	171 (10.1)	N.A.	
BMI (kg/m^2^)	25.5 (5.0)	N.A.	
Asthma and atopy characteristics			
Years since asthma diagnosis (rank 1–5)	4 3, 5	N.A.	
Number of positive aeroallergens (max 9)	4 3, 5	4 3, 4, 5	.989
Positive to a food allergen	44/158 (28)	36/158 (23)	.365
Positive to a seasonal allergen	120/158 (76)	113/158 (72)	.801
F_E_NO (ppb; Geometric mean [CI])	22.1 (20.0–24.5)	21.2 (19.2–23.4)	.286
S‐ECP (μg/L)	12.8 [7.30, 19.7]	12.7 [7.74, 19.55]	.461
Budesonide equivalent ICS dose (μg/day)	400 [400, 800]	400 [200, 800]	.431
Patients on LTRA	4/158 (2.5)	35/158 (22)	<**.001**
ACQ	0.83 [0.33, 1.33]	0.67 [0.17, 1.17]	**.015**
mAQLQ	5.94 [5.19, 6.51]	6.27 [5.70, 6.67]	<**.001**
GQLI	5.39 [4.94, 5.83]	5.33 [4.89, 5.83]	.674
FEV1 (% predicted)	79.0 (7.07)	79.8 (7.08)	.167

Data are median (IQR), mean (SD), or n/N (%), unless otherwise indicated. *n* = 158.

BMI, body mass index; years since asthma diagnosis (rank 1–5): 0–2 years = 1, 3–5 *y* = 2, 6–10 *y* = 3, 11–20 *y* = 4, >20 *y* = 5. Analysed allergens = cat, dog, birch, timothy, horse, mite (two), mugwort, and cladosporium (mold); Positive = IgE ≥ 0.35 kU_A_/L; F_E_NO, fraction of exhaled nitric oxide; S‐ECP, serum eosinophil cationic protein; ICS, inhaled corticosteroid; LTRA, leukotriene‐receptor antagonist; ACQ, asthma control questionnaire; mAQLQ, mini asthma quality of life questionnaire; GQLI, Gothenburg quality of life instrument; FEV_1_, forced expiratory volume in 1 sec; N.A., not applicable.

### Changes in IgE and IgG_4_ over one year

**Figure 1:**
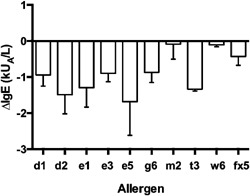
Absolute reductions in IgE‐antibody concentrations over one year. Arithmetic means and SEM. All reductions are significant (*P* < 0.05) except for w6 (data were converted to the log base 10 scale and analyzed using a paired *t*‐test). For allergen explanations, *P* values and relative changes, see Table 2.

Mean concentrations for almost all IgE antibodies and total IgE decreased significantly between baseline and the one‐year follow up (Fig. 1). The medians for the relative decrease of IgE concentrations were 7.8–36.4%, with the majority between 10% and 20%, and approximately two thirds of the population showed a decrease in various IgE‐antibody concentrations (Table 2). There was a significant negative correlation between age and IgE concentrations both at baseline and at the end of study for perennial and all IgE antibodies (Table S4). Further, the relative change in food IgE over one year showed a significant positive correlation with age (Table S5), which indicates that the reduction in food IgE was larger for younger subjects, and a trend toward a similar relationship was seen for total IgE. No significant difference between men and women was found for changes in IgE concentrations (Table S6). IgG_4_‐antibody concentrations against cat and timothy did not show any significant change during the study (Table 3).

**Table 2:** IgE‐antibody concentrations at baseline and last visit with relative changes

IgE type (kU_A_/L)	Baseline	One‐year follow‐up	Subjects with reduced IgE (n/N [%])	Median relative change (%)	*P*
Mite, d1	3.84 (11.8)	2.90 (9.04)	100/158 (63.3)	−12.1	**.008**
Mite, d2	6.19 (21.3)	4.70 (15.6)	104/157 (66.2)	−14.3	**.020**
Cat, e1	10.3 (19.3)	8.97 (18.3)	116/158 (73.4)	−16.2	**.008**
Horse, e3	3.69 (8.42)	2.79 (6.12)	105/158 (66.4)	−13.3	**.006**
Dog, e5	8.18 (29.5)	6.49 (18.6)	107/158 (67.7)	−14.2	**.033**
Timothy, g6	5.99 (16.8)	5.12 (15.2)	106/150 (70.7)	−20.8	<**.001**
Birch, t3	9.42 (17.9)	8.08 (14.8)	108/158 (68.4)	−17.7	<**.001**
Mould, m2	0.40 (2.34)	0.31 (1.88)	92/136 (67.6)	−36.4	<**.001**
Mugwort, w6	0.48 (1.45)	0.37 (0.98)	100/158 (63.3)	−10.8	.079
Perennial IgE	32.6 (58.1)	26.2 (44.2)	120/158 (75.9)	−16.0	<**.001**
Seasonal IgE	15.9 (28.5)	13.6 (24.8)	115/158 (72.8)	−17.4	<**.001**
Food IgE (fx5)	2.07 (10.5)	1.64 (9.61)	92/158 (58.2)	−7.80	**.004**
All specific IgE	50.5 (74.3)	41.4 (59.5)	114/158 (72.2)	−15.7	<**.001**
Total IgE	263 (464)	236 (425)	108/158 (68.4)	−10.2	<**.001**

Perennial = cat, dog, horse, mite (x2), and cladosporium. Seasonal = birch, timothy, and mugwort.

Food = cow's milk protein, egg white, peanut, soy, wheat, and fish. All IgE = perennial + seasonal + fx5.

Data are shown as mean (SD) unless otherwise indicated. Statistics: Data were converted to the log base 10 scale and analyzed using a paired *t*‐test.

**Table 3:** Changes in IgG_4_ concentrations over one year

IgG4 type (mg/L)	Baseline	One‐year follow‐up	*P*
Cat, e1	0.21 (0.16–0.27)	0.21 (0.17–0.27)	.936
Timothy, g6	0.10 (0.08–0.13)	0.10 (0.08–0.13)	.898

Data are shown as Geometric means (95%CI). *n* = 157. IgG4, immunoglobulin G4.

Statistics: Data were converted to the log base 10 scale and analyzed using a paired *t*‐test.

### Changes in IgE in relation to inflammatory markers

F_E_NO at baseline correlated significantly with concentrations of perennial and all IgE antibodies as well as those of total IgE, but not of seasonal and food IgE (Table S7). These relations were not consistent at last visit except for perennial IgE. There was a positive correlation between the reduction in F_E_NO and relative reduction in IgE concentrations for perennial IgE, all IgE and total IgE, and a trend for seasonal IgE (Table 4). When comparing subgroups that moved to or from the normal range of F_E_NO (<20 ppb) between baseline and last visit, we observed a significant difference in the change in seasonal and all IgE as well as total IgE, and a trend for perennial IgE (Table S8). The change in S‐ECP showed a positive correlation with the change in perennial IgE only (Table 4).

**Table 4:** Correlation analysis between relative change in IgE concentrations and changes in F_E_NO and S‐ECP or amount of treatment over one year

	Δ Perennial IgE		Δ Seasonal IgE		Δ Food IgE		Δ Total IgE		Δ All specific IgE	
	rho	*P*	rho	*P*	rho	*P*	rho	*P*	rho	*P*
Δ F_E_NO	0.23	**.003**	0.15	.058	0.09	.250	0.27	<**.001**	0.25	**.002**
Δ ECP	0.20	**.014**	0.02	.786	0.07	.376	0.08	.319	0.07	.377
Mean ICS dose	−0.17	**.030**	‐0.02	.789	−0.13	.103	−0.10	.194	−0.16	**.044**
Months of LTRA use	−0.20	**.013**	‐0.001	.903	−0.21	**.009**	−0.16	**.044**	‐0.17	**.036**

IgE, immunoglobulin E; F_E_NO, fraction of exhaled nitric oxide; ICS, inhaled corticosteroid; months of LTRA use, number of months with leukotriene receptor antagonist treatment.

Statistics: Spearman's rank correlation test.

### Changes in IgE in relation to use of anti‐inflammatory treatment

**Figure 2:**
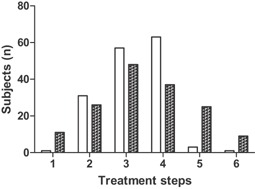
Distribution of treatment steps among study subjects at baseline (open bars) and last visit (filled bars). The distributions are significantly different (*P* < .001; *χ* ^2^ test).

Mean ICS use was 559 μg/day during the study and did not change significantly between baseline and last visit; however, the number of LTRA users increased significantly (Table 1). There was a significant change in the distribution of treatment steps between baseline and last visit, with more patients receiving the two highest doses of treatment or no treatment at all, and fewer patients at intermediate doses, at last visit compared to baseline (Fig. 2). Total LTRA use correlated significantly with the reduction of all groups of IgE except seasonal IgE, whereas mean daily ICS dose during the year showed a negative correlation with the relative change in perennial IgE and all IgE (Table 4).

### Relationships between changes in IgE, asthma control, and quality of life

There was a significant correlation between improvements in the ACQ and mAQLQ scores and the relative reduction in total IgE as well as perennial and all IgE‐antibody groups (Table 5). When comparing subjects that showed a clinically important improvement in ACQ score (>0.5) with all other subjects, a significant difference in the reduction in perennial and all IgE‐antibody groups as well as total IgE was seen (Table 6). Results for separate mAQLQ domains are shown in Table S9. No significant correlations with change in the GQLI score were seen. The change in F_E_NO showed a weak correlation with change in ACQ score only (Table 5).

**Table 5:** Correlation analysis between relative change in IgE concentrations and F_E_NO, and change in ACQ, mAQLQ, and GQLI over one year

	Δ F_E_NO		Δ Perennial IgE		Δ Seasonal IgE		Δ Food IgE		Δ Total IgE		Δ All specific IgE	
	rho	*P*	rho	*P*	rho	*P*	rho	*P*	rho	*P*	rho	*P*
Δ ACQ	0.16	**.046**	0.21	**.012**	0.15	.069	0.12	.159	0.24	**.004**	0.19	**.019**
Δ mAQLQ	−0.12	.148	−0.21	**.009**	−0.10	.228	−0.12	.142	−0.17	**.034**	−0.16	**.042**
ΔGQLI	−0.03	.662	−0.01	.942	0.03	.698	−0.03	.707	−0.02	.796	−0.01	.946

ACQ, asthma control questionnaire; mAQLQ, mini asthma quality of life questionnaire; GQLI, Gothenburg quality of life instrument; Statistics, Spearman's rank correlation test.

**Table 6:** Median (IQR) change in IgE concentrations (kU_A_/L) in subjects with or without a clinically important improvement in ACQ score during the study

	Δ ACQ < 0.5 (*n* = 107)	Δ ACQ ≥ 0.5 (*n* = 48)	*P*
Perennial IgE	−0.88 (−4.01, −0.01)	−3.67 (−17.9, −0.73)	**.008**
Seasonal IgE	‐0.33 (−2.45, 0.05)	−0.29 (−3.11, −0.04)	.461
Food IgE	‐0.02 (−0.86, 0.10)	−0.06 (−1.09, 0.68)	.516
All IgE	‐2.17 (−8.70, 0.18)	−5.82 (−25.0, −1.32)	**.022**
Total IgE	‐7.05 (−18.1, 4.16)	−20.9 (−77.5, −1.00)	**.019**

### Analysis of the effect of allergen exposure

Thirty‐one participants were exposed daily to relevant pets (cat and dog) and 84 reported that they never had contact with pets during the study (Table S10). At the study start, 28 participants reported a pet at home and six of them removed their pet during the study. Two participants got a pet during the study. The proportion of participants with pets at home compared with those who reported no contact with pets did not change significantly between baseline and last visit (*P* = .443). Concentrations of IgE antibodies against pets were significantly higher both at the beginning and end of the study in those who had daily contact with pets than in those who never had contact. However, both groups significantly lowered their IgE concentrations for pets during the study, with no significant difference between the groups (Table S11). Further, there was no difference in the proportion of patients reporting dampness in the home between baseline and last visit (*P* = .27).

There was no major difference in reported pollen levels between the years of the study, except that birch pollen were high in 2006 (Table S12, Fig. S1). Fifteen participants were included during the year after the heavy birch pollen season 2006. These participants did not have higher IgE concentration for birch at inclusion than those who were included after the birch pollen season 2007 and onwards, but did have a significantly larger reduction of IgE against birch during the study, although both groups showed a significant reduction in birch IgE concentration (Table S11). However, there was no significant difference between the changes in other types of IgE between these two groups (Table S13). Analysis of the influence of the time of year when a patient was included in the study revealed a significant difference for seasonal and food IgE but not for the other IgE groups or total IgE. Those included during quarter two (April–June) had a significantly smaller reduction in seasonal IgE compared to those included during quarter four (October–December) and a smaller reduction in food IgE compared to those included during quarter three (July–September) (*P* < .01 both comparisons).

## Discussion

This one‐year follow‐up study in adult patients with atopic asthma showed that optimized anti‐inflammatory treatment with inhaled corticosteroids and a leukotriene‐receptor‐antagonist resulted in a significant decrease in both total IgE and IgE‐antibody concentrations while exposure to relevant allergens was essentially unchanged. The amount of ICS treatment received during the year correlated significantly with the reduction in IgE antibodies to perennial allergens, the type of sensitization with the greatest impact on airway inflammation. Further, the total use of LTRA significantly correlated with the reduction in food IgE and total IgE, as well as perennial IgE. The effect on IgE seemed to be related to a reduction in F_E_NO and, to a lesser extent, to changes in serum ECP. Interestingly, the decrease in IgE concentrations, especially IgE to perennial allergens, correlated with improvement in both asthma control and asthma‐related quality of life.

The major finding of our study was a substantial decrease in IgE concentrations, with relative reductions of 10–20% for most IgE antibodies measured, as well as for total IgE. IgE to mold showed the largest relative reduction, but the mean baseline IgE‐antibody concentration was low for this allergen. Interestingly, not only IgE to aeroallergens but also to food allergens was significantly reduced in this study. We chose to include IgE to food in the analysis since a large proportion of adult asthmatics seem generally to be sensitized to food 17, and we confirmed food sensitization in almost 30% of the subjects in our material. Further, such sensitization has recently been shown to independently associate with markers of type‐2 immunity (F_E_NO and blood eosinophil count) in asthmatics 18. The reduction in IgE is most probably related to an optimization of the anti‐inflammatory treatment through a redistribution of treatment steps (13). In other words, a larger proportion of the participants were at steps with either the highest or the lowest level of anti‐inflammatory treatment at the end of the study, as compared to baseline. Available data on the natural evolution of IgE concentrations in adults are contradictory as discussed by Patelis et al. 19. However, one large population‐based study (*n* = 2300) in adults 30–40 years of age showed unchanged prevalence of IgE sensitization to basically all aeroallergens and increased total IgE concentrations over a 9‐year follow‐up period 19. Thus, there is little support for the view that the changes in IgE we see in the present one‐year follow‐up study would be due to natural changes.

Very few data on changes in IgE concentrations due to treatment with anti‐inflammatory drugs are available, and the results of earlier studies have been contradictory. In the 1970s, a transient increase of IgE concentrations, followed by a decrease to baseline or below, was described in children and adults with mainly steroid‐naïve atopic asthma who were treated with a short course of oral corticosteroids 7, 8. The transient increase of IgE did not seem to cause any worsening of asthma 9. The reason for this increase can only be speculated upon, but may be related to a rapid decrease in IgE‐binding sites, resulting in increased levels of free measurable IgE antibodies. More recent studies on treatment‐naïve patients with atopic asthma or food allergy indicate that the introduction of anti‐inflammatory treatment with at least medium doses of ICS, as well as treatment with LTRA, reduces IgE concentrations 10, 11, 20. In contrast, low dose ICS has repeatedly been shown not to reduce IgE 10, 11, 21, or even to cause a transient increase in IgE concentrations 11. This is the first long‐term study to show an effect on IgE concentrations in patients with asthma who were on ICS treatment at baseline, brought about simply by optimizing the anti‐inflammatory treatment (ICS and LTRA) according to F_E_NO or symptoms.

Interestingly, the reduction in relevant groups of IgE antibodies and total IgE correlated with improvement in asthma symptom control, measured with ACQ and the symptom domain of mAQLQ. More importantly, the reduction in IgE was significantly larger in subjects showing a clinically important change in ACQ score. Thus, control of IgE formation may be related to asthma symptom control. Furthermore, improvement in asthma‐related quality of life correlated with reductions in relevant IgE‐antibody groups and total IgE, but not with changes in F_E_NO. This may be due to the fact that reductions in IgE, but not F_E_NO, related to improvements in the environmental (trend) and emotional domains of the mAQLQ instrument.

We could for the first time show a significant correlation between the change in F_E_NO and the relative change of important groups of IgE antibodies and total IgE. Serum ECP, which signals systemic eosinophilic inflammation, correlated only with the relative change of perennial IgE antibodies. However, blood eosinophil count may be a better marker of (IgE‐mediated) systemic Th2‐driven inflammation 22, 23, as ECP levels may be influenced by virus exposure 24, 25. The correlation between changes in IgE concentrations and changes in F_E_NO seemed stronger than that between anti‐inflammatory drug use and changes in IgE. This is reasonable since the confirmed anti‐inflammatory effect (reduction in F_E_NO) should be more important for effects on IgE levels than the amount of drug given per se. From a mechanistic point of view, it is reasonable to suggest that anti‐inflammatory treatment with ICS and LTRA can reduce IgE synthesis since both agents inhibit the Th2 lymphocyte, resulting in reduced secretion of IL‐4, IL‐13, and IL‐5 5, 26, 27, 28, 29. Airway NO is produced by inducible NO synthase (iNOS) in the bronchial epithelium and the expression of iNOS is up‐regulated by IL‐4 and IL‐13 5. Both ICS and LTRA treatment probably reduce F_E_NO through reduction of IL‐4 and IL‐13 release 5. Furthermore, data from experimental models suggest that the major part of IgE synthesis takes place in or near the organ which is in contact with the allergen 30. This could explain why ICS treatment mainly seemed to affect the synthesis of aeroallergen IgE in our study, with no effect on food IgE, while LTRA treatment related to an effect on both these groups of IgE antibodies. Further, FeNO correlated with IgE levels at baseline but this correlation had basically disappeared at the follow up. This indicates that a change in anti‐inflammatory treatment causes a relatively rapid effect on inflammation (FeNO) whereas the effect on IgE is slower, and, thus, leading to disruption of the correlation. Taken together, it is suggested that the treatment‐related long‐term reduction of IL‐4 and IL‐13, signalled by lowered F_E_NO, leads to reduced formation of IgE 5.

A strength of the present study is that all paired serum measurements were analyzed side‐by‐side in the instrument. Another strength is the thorough collection of data on both outdoor (grass and tree pollen) and indoor (furry animal, dampness in the home) allergen exposure. These analyses showed very consistent exposure levels over the study years, with the exception of high birch pollen counts during the spring of 2006. This extreme season slightly strengthened the effect on IgE to birch pollen over one year but did not affect the reduction of IgE to the other allergens or total IgE. Furthermore, IgG_4_ antibodies to the two common aeroallergens cat and timothy did not change at all during the study. The concentration of IgG_4_ antibodies has been shown to correlate with the degree of cat allergen exposure in the home regardless of the degree of IgE sensitization 31, and a reduction in exposure to house‐dust mite has been shown to result in reduced IgG_4_‐antibody concentrations 32. However, subjects included during the tree pollen season (April–June) showed a smaller reduction in seasonal and food IgE than those included during the autumn. This indicates that tree pollen exposure counteracted the drug treatment‐related effect on IgE to pollen allergens and cross‐reactive food allergens, without affecting the reduction in perennial IgE antibodies or total IgE. All in all, our data strongly support the view that a change in allergen exposure does not explain the reductions in IgE that we see in our study. A weakness of the study may be that the patients included in this analysis have followed two different treatment protocols. However, the treatment options were the same in these groups and the only difference was the basis for a decision to change treatment: F_E_NO or symptoms. Thus, those with low F_E_NO or fewer/milder asthma symptoms received less treatment and those with high F_E_NO or more/stronger symptoms increased their use of ICS and LTRA. As a result, participants in both study groups with a high baseline F_E_NO increased their LTRA and ICS use, and F_E_NO decreased significantly in these patients without any significant difference between the two groups. A relationship between a change in F_E_NO and a change in IgE concentrations was seen at the individual level in this study. However, whereas IgE concentrations were significantly reduced at the group level, F_E_NO was not. This may seem contradictory but could be explained by the fact that the overall ICS use did not change during the study while LTRA use increased significantly. It is well known that F_E_NO is strongly affected by ICS treatment but much less so by LTRA treatment 5. At the same time, both these agents seem able to reduce IgE formation 10, 11, 18, which could explain the present findings at the group level. Furthermore, it must be noted that we report that the reduction in IgE correlated with the total use of ICS and LTRA during the study, and not the change in drug use.

In conclusion, this one‐year follow‐up study on relatively well‐controlled adults with atopic asthma and ongoing inhaled corticosteroid treatment at baseline, using merged data from a randomized, controlled trial, showed that optimized treatment with inhaled corticosteroids and a leukotriene‐receptor antagonist resulted in a marked decrease in both total IgE and IgE‐antibody concentrations. The decrease in total IgE and perennial IgE‐antibody concentration correlated with reductions in F_E_NO as well as with improvements in asthma control and asthma‐related quality of life. While F_E_NO signals short‐term (days and weeks) changes in Th2‐driven airway inflammation, the IgE concentration may reflect more long‐term positive effects (months and years) of anti‐inflammatory treatment that are clinically important.

## Conflicts of Interest

KA has received research support from Aerocrine AB and Thermo Fisher Scientific. MB is an associate of Thermo Fisher Scientific. JS has received research support from Aerocrine AB. AM, ALU, AA, and ML declare no conflict of interest.

## Supporting information

Additional supporting information may be found in the online version of this article at the publisher's web‐site.

**Table S1**. Treatment steps allowed in the study.
**Table S2**. Study design.
**Table S3**. Change in IgE in the two original study groups.
**Table S4**. Correlation analysis between age and IgE concentrations at baseline and last visit.
**Table S5**. Correlation analysis between age and relative change in IgE concentrations over one year.
**Table S6**. Comparison of gender and relative change of IgE concentrations over one year.
**Table S7**. Correlation analysis between IgE concentrations and F_E_NO at baseline and last visit.
**Table S8**. Median (IQR) change in IgE concentrations (kU_A_/L) in subgroups moving up or down between the normal and elevated range of F_E_NO during the study (cut‐off 20 ppb).
**Table S9**. Correlation analysis between relative change in IgE concentrations and change in mAQLQ domain scores over one year.
**Table S10**. Exposure to pets during the study.
**Table S11**. Comparison of change in IgE concentrations after different exposures to pets and birch pollen.
**Table S12**. Sum of AUC for different pollens in Stockholm, Forshaga, and Malmö.
**Table S13**. Median relative change in IgE concentrations over one year.
**Figure S1**. Birch pollen count in Stockholm during the spring, 2006 and 2007. AUC, area under curve.
