# Supplementary material for: Parallel reductions of IgE and exhaled nitric oxide after optimized anti‐inflammatory asthma treatment
Source: Immun Inflamm Dis. 2016 Mar 21;4(2):182–90. doi: 10.1002/iid3.103 (PMC4879464; doi:10.1002/iid3.103)
Supplement: Supplementary file 1 — Table S1. Treatment steps allowed in the study. Table S2. Study design. Table S3. Change in IgE in the two original study groups. Table S4. Correlation analysis between age and IgE concentrations at baseline and last visit. Table S5. Correlation analysis between age and relative change in IgE concentrations over one year. Table S6. Comparison of gender and relative change of IgE concentrations over one year. Table S7. Correlation analysis between IgE concentrations and FENO at baseline and last visit. Table S8. Median (IQR) change in IgE concentrations (kUA/L) in subgroups moving up or down between the normal and elevated range of FENO during the study (cut‐off 20 ppb). Table S9. Correlation analysis between relative change in IgE concentrations and change in mAQLQ domain scores over one year. Table S10. Exposure to pets during the study. Table S11. Comparison of change in IgE concentrations after different exposures to pets and birch pollen. Table S12. Sum of AUC for different pollens in Stockholm, Forshaga, and Malmö. Table S13. Median relative change in IgE concentrations over one year. Figure S1. Birch pollen count in Stockholm during the spring, 2006 and 2007. AUC, area under curve. [file IID3-4-182-s001.docx]

**Supporting Information**

**Methods**

In the original study, strict FeNO-guided management (and not symptoms) was compared with usual care (primarily based on symptoms and short-acting bronchodilator use according to national guidelines at the time). Since it would not be logical to step up treatment with long-acting bronchodilators based on elevated FeNO, we wanted to use only anti-inflammatory treatment in the study and therefore long-acting bronchodilators were excluded. Patients did not receive a written asthma plan since the control arm was usual care in this primary care study, and written asthma plans were not part of standard of care at the time of the study. Inhalation technique was checked in all patients at baseline.

Pollen count data for 2006 to 2009 were collected from three measuring stations: Stockholm, Forshaga and Malmö. Data from Malmö were provided by Pollen Laboratory, Gothenburg Atmospheric Science Center, Department of Biological and Environmental Sciences, University of Gothenburg, and data from Stockholm and Forshaga were from Palynological Laboratory, Swedish Museum of Natural History, Stockholm. At these stations, air is drawn continuously , at a rate of 10 litres/minute, into a “Burkard Seven Day Volumetric Spore Trap” where pollen gets caught on adhesive tape. The tape is manually examined under a microscope on a daily basis, and the result is expressed as “pollen particle count/m^3^ air/day”. Daily results can be plotted in a diagram and the total seasonal impact for an allergen can be estimated by calculating the area under the curve (AUC) (see figure E1). See http://www.nrm.se/english/researchandcollections/environmentalresearchandmonitoring/palynologicallaboratory.7000_en.html for more information (Swedish Museum of Natural History).

F_E_NO was measured with a standardised method according to ATS/ERS guidelines. Participants inhaled to total lung capacity and then immediately exhaled into the NO analyser (NIOX MINO^®^) for 10 seconds at a flow rate of 50 ml/s. The mean of two measurements was used. A third measurement was added if the two first differed >10%.

Environmental exposure was assessed with the following questions:

1. Do you have any pet animal at home? – if yes, what sort of animal?

2. Are you regularly (at least once a week going back at least one month) exposed to furred animals outside your home? – if yes, what sort of animal?

3. Have you, in last two weeks, been exposed (more than usual) to anything you know you are allergic to? – if yes, when? What type of allergen? How long were you exposed?

4. Do the window panes in your home show condensation on the inside during the winter?

The Gothenburg Quality of Life Instrument (GQLI) was used to assess general well-being (social, physical and mental). Social well-being was rated with questions about: home and family situation, housing, work situation, economy, health and leisure. Physical well-being was rated with questions about: hearing, vision, memory, fitness and appetite. Mental well-being was rated with questions about: mood, energy, patience, self-confidence and sleep [16]. All items were rated on a Likert 7-point scale from “very poor” (1 point) to “excellent, could not be better” (7 points).

Elizabeth Juniper’s mini-Asthma Quality of Life Questionnaire (mAQLQ) was used to assess asthma-related quality of life. There are 15 questions in the mAQLQ within 4 different domains (symptoms – 5 items, activity limitation – 4 items, emotional function – 3 items, and environmental stimuli – 3 items). Participants score their experience during the past two weeks on a 7-point scale (7 = not impaired at all – 1 = severely impaired). The overall mAQLQ score is the mean of all 15 responses and the individual domain scores are the means of the items in those domains. A change in the score of 0.5 is considered the minimal clinically important difference. No cut-off defining poor asthma-related quality of life has been set.

Elizabeth Juniper’s Asthma Control Questionnaire (ACQ) with six questions (FEV_1_% predicted was not included) was used to assess asthma control. Participants score their experience during the past week on a 7-point scale (0=no impairment, 6=maximum impairment). The ACQ score is the mean of the six questions. A change in the score of 0.5 is considered the minimal clinically important difference. A score of >1.25 or >1.5 can be used to define asthma that is not under good control; both score cut-offs have high positive predictive value. We used ACQ and mAQLQ with written permission from Professor Elizabeth Juniper.

**Table E1.** Treatment steps allowed in the study

| **Dose step** | **1** | **2** | **3** | **4** | **5** | **6** | |  |
| --- | --- | --- | --- | --- | --- | --- | --- | --- |
| Budesonide (µg/day) | 0 | 200 | 400 | 800 | 800 + LTRA | | 1600 + LTRA | |
| Fluticasone (µg/day) | 0 | 100 | 250 | 500 | 500 + LTRA | | 1000 + LTRA | |
| Mometasone (µg/day) | 0 | 100 | 200 | 400 | 400 + LTRA | | 800 + LTRA | |

LTRA = leukotriene receptor antagonist (montelukast 10 mg daily)

**Table E2.** Study design

|  | **Visit 1**  **Screening** | **Visit 2**  **Baseline** | **Visit 3**  **2 months** | **Visit 4**  **4 months** | **Visit 5**  **8 months** | **Visit 6**  **12 months** |
| --- | --- | --- | --- | --- | --- | --- |
| ImmunoCAP Rapid  Capillary blood test  Eligibility check for inclusion | X |  |  |  |  |  |
| F_E_NO |  | X | X | X | X | X |
| Spirometry with reversibility test.  Venous blood sample. |  | X |  |  |  | X |
| mAQLQ, GQLI |  | X |  | X |  | X |
| ACQ |  | X | X | X | X | X |
| Registration of exacerbations, SABA and ICS use. Questions about exposure to pets |  | X | X | X | X | X |

F_E_NO = fraction of exhaled nitric oxide, mAQLQ = mini asthma quality of life questionnaire,

GQLI = Gothenburg quality of life instrument, ACQ = asthma control questionnaire, ICS = inhaled corticosteroid, SABA = short-acting beta agonist.

**Table E3.** Change in IgE in the two original study groups

| Variables | Groups | Δ IgE, median relative change (%) | Between groups* | Between visit 1 and 5** |
| --- | --- | --- | --- | --- |
| Perennial IgE | F_E_NO-guided | -19.4 | p=.93 | p=.019 |
|  | Control | -14.7 |  | p<.001 |
| Seasonal IgE | F_E_NO-guided | -14.1 | p=.73 | p=.039 |
|  | Control | -16.2 |  | p=.002 |
| Food IgE | F_E_NO-guided | -9.4 | p=.82 | p=.044 |
|  | Control | -7.8 |  | p=.044 |
| Total IgE | F_E_NO-guided | -10.3 | p=.66 | p=.013 |
|  | Control | -10.1 |  | p=.013 |

Statistics: * Mann-Whitney U test. **IgE data were converted to the log base 10 scale and analysed with paired

*t*-test.

**Table E4.** Correlation analysis between age and IgE concentrations at baseline and last visit

|  | **Perennial IgE** | | **Seasonal IgE** | | **Food IgE** | | **Total IgE** | | **All specific IgE** | |
| --- | --- | --- | --- | --- | --- | --- | --- | --- | --- | --- |
|  | rho | p | rho | p | rho | p | rho | p | rho | p |
| **Baseline** |  |  |  |  |  |  |  |  |  |  |
| Age | -0.17 | **.032** | -0.15 | .053 | -0.04 | .581 | -0.01 | .986 | -0.22 | **.006** |
| **One-year follow-up** |  |  |  |  |  |  |  |  |  |  |
| Age | -0.19 | **.017** | -0.13 | .094 | 0.02 | .843 | 0.03 | .713 | -0.21 | **.009** |

Statistics: Spearman’s rank correlation test. N=158.

**Table E5.** Correlation analysis between age and relative change in IgE concentrations over one year

|  | **Δ Perennial IgE** | | **Δ Seasonal IgE** | | **Δ Food IgE** | | **Δ Total IgE** | | **Δ All specific IgE** | |
| --- | --- | --- | --- | --- | --- | --- | --- | --- | --- | --- |
|  | rho | p | rho | p | rho | p | rho | p | rho | p |
| Age | 0.07 | .401 | 0.13 | .100 | 0.17 | **.032** | 0.15 | .058 | 0.12 | .144 |

Statistics: Spearman’s rank correlation test. N=158.

**Table E6.** Comparison of gender and relative change of IgE concentrations over one year

| Median relative change (%) | | | | | | | | | | | |
| --- | --- | --- | --- | --- | --- | --- | --- | --- | --- | --- | --- |
|  | **n** | **Perennial IgE** | **p** | **Seasonal IgE** | **p** | **Food IgE** | **p** | **Total IgE** | **p** | **All spec. IgE** | **p** |
| Men | 82 | -14.2 | .429 | -17.4 | .914 | -5.52 | .402 | -6.82 | .384 | -15.4 | .417 |
| Women | 76 | -18.7 |  | -16.9 |  | -7.90 |  | -12.8 |  | -15.7 |  |

Statistics: Mann-Whitney U test. Data are shown as median [IQR].

**Table E7.** Correlation analysis between IgE concentrations and F_E_NO at baseline and last visit

| **F_E_NO** | **Baseline** | | **One-year follow-up** | |
| --- | --- | --- | --- | --- |
|  | rho | p | rho | p |
| Perennial IgE | 0.28 | **<.001** | 0.21 | **.007** |
| Seasonal IgE | 0.07 | .356 | -0.07 | .361 |
| Food IgE | 0.11 | .186 | 0.06 | .423 |
| All specific IgE | 0.27 | **<.001** | 0.15 | .054 |
| Total IgE | 0.26 | .**001** | 0.08 | .298 |

Statistics: Data were converted to the log base 10 scale and analysed with Pearson’s correlation test. N=158.

**Table E8.** Median [IQR] change in IgE concentrations (kU_A_/L) in subgroups moving up or down between the normal and elevated range of F_E_NO during the study (cut-off 20 ppb).

|  | Normal to elevated  (n=25) | Elevated to normal  (n=34) |  |
| --- | --- | --- | --- |
| F_E_NO (ppb, Geometric mean) | 15 to 33 ppb | 32 to 15 ppb | p |
|  |  |  |  |
| Perennial IgE | -1.25 [-5.13, 0.06] | -2.23 [-12.6, -0.73] | 0.07 |
| Seasonal IgE | -0.06 [-1.41, 0.09] | -0.57 [-6.88, -0.07] | **0.03** |
| Food IgE | -0.01 [-0.29, 0.11] | -0.19 [-6.13, 0.03] | 0.13 |
| All IgE | -2.10 [-5.56, 0.63] | -5.41 [-32.5, -0.77] | **0.02** |
| Total IgE | -6.89 [-18.3, 19.6] | -18.8 [-73.9, -1.02] | **0.02** |
|  |  |  |  |

**Table E9.** Correlation analysis between relative change in IgE concentrations and change in mAQLQ domain scores over one year

|  | **Δ F_E_NO** | | **Δ Perennial** | | **Δ Seasonal** | | **Δ Food** | | **Δ Total** | | **Δ Sum all specific** | | | | |  |
| --- | --- | --- | --- | --- | --- | --- | --- | --- | --- | --- | --- | --- | --- | --- | --- | --- |
|  | rho | p | rho | p | rho | p | rho | p | rho | p | | rho | | | p | |
| Δ AQLQ Symptoms | -0.14 | .082 | -0.29 | **<.001** | -0.15 | .063 | -0.12 | .131 | -0.25 | **.001** | | | -0.24 | **.003** | |  |
| Δ AQLQ Activity | -0.09 | .250 | -0.08 | .318 | -0.02 | .813 | -0.02 | .789 | -0.08 | .312 | | | -0.08 | .343 | |  |
| Δ AQLQ Emotional | -0.13 | .107 | -0.19 | **.016** | -0.04 | .629 | -0.09 | .260 | -0.16 | **.049** | | | -0.15 | .063 | |  |
| Δ AQLQ Environmental | -0.07 | .392 | -0.11 | .184 | -0.10 | .200 | 0.01 | .891 | -0.06 | .413 | | | -0.13 | .100 | |  |

Statistics: Spearman rank correlation test. N=158

**Table E10.** Exposure to pets during the study

|  | **Always** | **Sometimes** | **Never** |
| --- | --- | --- | --- |
| Pet at home | 13.9 | 7.6 | 78.5 |
| Exposure to pet outside home | 7.6 | 34.2 | 58.2 |
| Exposure to pet at home and/or outside home | 19.6 | 27.2 | 53.2 |
| Data are n/N (%). N=158 |  |  |  |

**Table E11.** Comparison of change in IgE concentrations after different exposures to pets and birch pollen

|  | **n** | **Pet IgE at baseline** | **p #** | **Pet IgE at**  **1-year follow-up** | **p #** | **Relative change of pet IgE** | **p #** | **p ##** |
| --- | --- | --- | --- | --- | --- | --- | --- | --- |
| Always exposure to pets | 31 | 9.85 (5.96-16.3) | **.001** | 8.95 (5.58-14.3) | **.001** | -22.0 [-37.5-6.29] | .184 | **.023** |
| Never exposure to pets | 84 | 2.33 (1.42-3.82) |  | 2.10 (1.28-3.45) |  | -12.6 [-25.4- -0.01] |  | **<.001** |
|  |  | **Birch IgE at baseline** |  | **Birch IgE at 1-year follow-up** |  | **Relative change of Birch IgE** |  |  |
| Inclusion before birch season 2007 | 15 | 1.00 (0.24-4.14) | .771 | 0.60 (0.14-2.58) | .837 | -39.1 [-48.6- -24.8] | **<.001** | **<.001** |
| Inclusion after birch season 2007 | 143 | 0.80 (0.49-1.30) |  | 0.71 (0.43-1.16) |  | -14.9 [-29.9-19.1] |  | **<.001** |

Pet IgE = sum of IgE antibodies to dog and cat, # = between groups, ## = between visits.

Statistics: Data are shown as Geometric means (95% CI) and median [IQR]. IgE data were converted to the log base 10 scale and analysed with Student’s *t*-test. Wilcoxon signed-rank test and Mann-Whitney U test were used for analysis of relative change of IgE.

**Table E12.** Sum of AUC for different pollens in Stockholm, Forshaga and Malmö

| **Pollen** | **2006** | **2007** | **2008** | **2009** |
| --- | --- | --- | --- | --- |
| Tree | 77.451 | 32.524 | 30.475 | 42.199 |
| Grass | 5.059 | 6.399 | 6.492 | 5.970 |
| Mugwort | 822 | 912 | 587 | 820 |
| Sum | 83.332 | 39.835 | 37.554 | 48.989 |

Unit of measure = Pollen particle count/m^3^ air/day. AUC=Area under curve.

**Table E13.** Median relative change in IgE concentrations over one year

|  | **Inclusion before birch season 2007 (n=15)** | **Inclusion after birch season 2007 (n=143)** |  |  |
| --- | --- | --- | --- | --- |
| Variables | Median relative change (%) | | p |  |
| Mite, d1 | 2.04 | -12.7 | .087 |  |
| Mite, d2 | -9.43 | -16.1 | .487 |  |
| Cat, e1 | -15.8 | -16.4 | .881 |  |
| Horse, e3 | -14.3 | -12.9 | .770 |  |
| Dog, e5 | -10.7 | -15.8 | .960 |  |
| Timothy, g6 | -18.9 | -21.1 | .193 |  |
| Birch, t3 | -39.1 | -14.9 | **<.001** |  |
| Mould, m2 | -34.2 | -36.8 | .847 |  |
| Mugwort, w6 | -16.1 | -10.4 | .840 |  |
| Perennial IgE | -15.1 | -16.1 | .620 |  |
| Seasonal IgE | -28.8 | -16.0 | .038 |  |
| Food IgE | -11.6 | -7.35 | .856 |  |
| All IgE | -13.5 | -9.63 | .074 |  |
| Total IgE | -22.6 | -14.7 | .571 |  |

Statistics: IgE data were converted to the log base 10 scale and analysed with paired *t*-test.


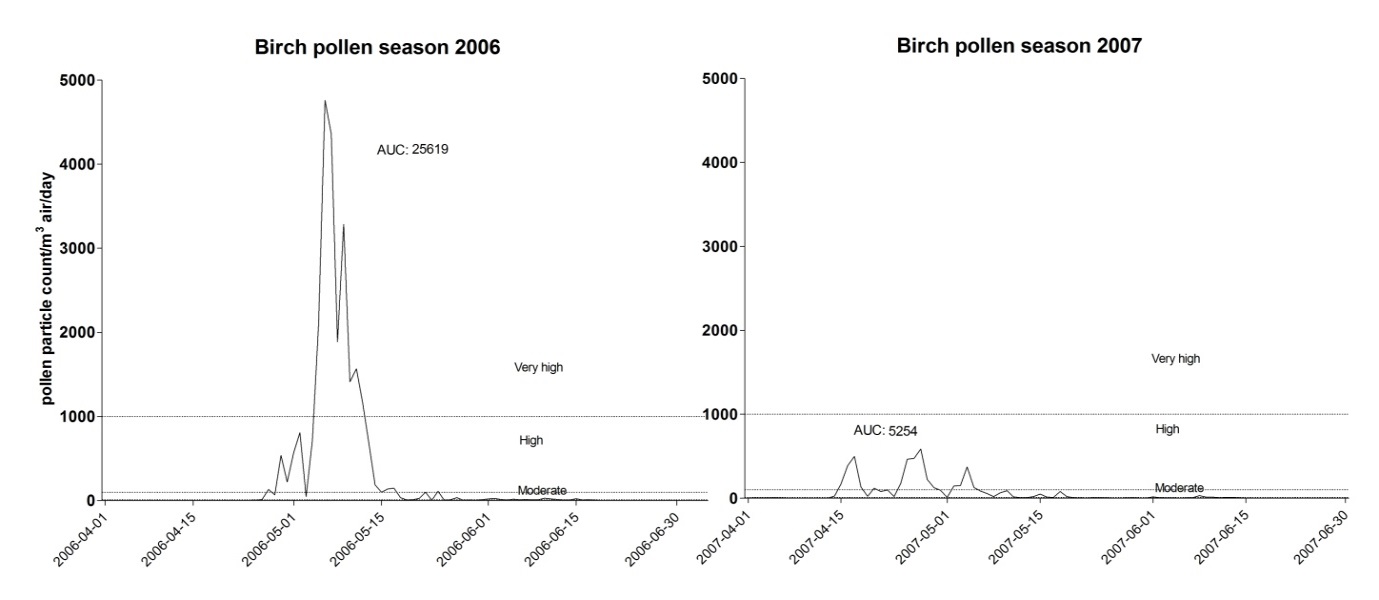


**Figure E1.** Birch pollen count in Stockholm during the spring, 2006 and 2007. AUC=Area under curve.
